# Oral Health, Anxiety, Depression, and Stress in Pregnancy: A Rapid Review of Associations and Implications for Perinatal Care

**DOI:** 10.3390/ijerph22010032

**Published:** 2024-12-29

**Authors:** Abiola A. Adeniyi, Swathi Ramachandran, Cecilia Marie Jevitt

**Affiliations:** 1 School of Policy and Global Studies, Fairleigh Dickinson University, Vancouver, BC V6B 2P6, Canada; abiola.adeniyi@ubc.ca; 2 University of British Columbia Centre for Disease Control, BC Centre for Disease Control, Vancouver, BC V5Z 4R4, Canada; swathir@student.ubc.ca; 3 Department of Family Practice, Midwifery Program, Faculty of Medicine, University of British Columbia, Vancouver, BC V6T 1Z3, Canada

**Keywords:** pregnancy, oral health, anxiety, depression, stress, biopsychosocial pathways, integrated prenatal care

## Abstract

Research demonstrates associations between oral health and specific mental health conditions in the general population, yet these relationships remain understudied during pregnancy, despite pregnancy’s profound effects on both oral and psychological well-being. Our rapid review examines current evidence on associations between oral health conditions and psychological states (anxiety, depression, and stress) during pregnancy, aiming to inform and strengthen integrated prenatal care strategies. Following PRISMA-RR guidelines, we conducted a systematic search on OVID Medline, CINAHL, and PsycINFO (January 2000–November 2024) for studies examining relationships between oral health conditions (periodontal disease, dental caries) and psychological status during pregnancy and up to one year postpartum. Systematic screening of 1201 records yielded 22 eligible studies (13 cross-sectional studies, 3 longitudinal cohort studies, 3 comparative studies, 2 prospective studies, and 1 case–control study). Analysis confirmed significant associations between oral health and psychological well-being during pregnancy through three pathways: psychological (dental anxiety directly limits oral healthcare utilization), behavioral (maternal depression reduces oral health self-efficacy), and physiological (elevated stress biomarkers correlate with periodontal disease, and periodontal therapy is associated with reduced salivary cortisol). These interactions extend intergenerationally, with maternal psychological distress showing significant associations with children’s caries risk. Evidence suggests interactions between oral health conditions and psychological states during pregnancy, warranting integrated care approaches. We recommend: (1) implementing combined oral–mental health screening in prenatal care, (2) developing interventions targeting both domains, and (3) establishing care pathways that address these interconnections. This integrated approach could improve both maternal and child health outcomes.

## 1. Introduction

Pregnancy causes major physiological and psychological changes that significantly affect oral and mental health [1]. Hormonal changes during pregnancy increase the risk of periodontal diseases, with gingivitis affecting 30–100% of pregnant individuals across different populations [2,3]. These changes occur through well-documented biological mechanisms, including increased levels of progesterone and estrogen that alter the inflammatory response and modify the oral microbiome composition [4]. The relationship between oral health and psychological well-being during pregnancy appears to operate through multiple pathways [5]. Current evidence suggests three primary mechanisms: (1) neuroendocrine pathways, where stress hormones like cortisol affect immune function and inflammatory responses [6,7], (2) behavioral pathways, where psychological distress impacts oral hygiene practices and healthcare utilization [8], and (3) immune–inflammatory pathways, where psychological stress modulates immune responses and periodontal inflammation [9,10].

A systematic review and a scoping review have highlighted the urgent need to integrate oral health into prenatal care [11,12]. Specific psychological challenges, including anxiety and depression [13], also increase during this period, with professional guidelines recommending integration of psychological assessment and support into routine prenatal care to address this increased psychological vulnerability [14]. While associations between oral health and psychological well-being are established in the general population [15,16,17], research examining these relationships during pregnancy remains limited. Recent evidence examining oral microbiome patterns in pregnancy found associations between oral bacterial communities and maternal anxiety and depression levels [18], suggesting biological mechanisms may exist, but studies investigating clinical oral health outcomes are sparse. This knowledge gap hinders effective care integration at a time when both domains undergo significant changes.

Understanding the relationship between oral and psychological well-being during pregnancy is particularly crucial given that socioeconomic factors often limit access to both oral and mental health care, perpetuating health inequalities [19,20,21]. If an association exists during pregnancy, identifying oral health issues early may serve as an indicator of overall health and potential psychological risks. While psychological screening is now more common in prenatal care, oral health assessment varies even though recommendations support its inclusion [11,12]. The consequences of failing to understand and address these interconnections may include adverse pregnancy outcomes. Research has shown associations between poor oral health and preterm birth, low birth weight, and increased risk of maternal complications [22,23]. Furthermore, poor oral health during pregnancy has been associated with increased inflammation and systemic health issues [24,25] while untreated psychological conditions can impact prenatal care adherence, nutrition, and overall maternal well-being [26,27]. Understanding these relationships could strengthen the case for comprehensive prenatal oral health screening, potentially improving both maternal psychological health and pregnancy outcomes [2,13].

To address this evidence gap, the team conducted a rapid scoping review which allows for timely decision making. This review examines the relationships between oral health and specific psychological conditions (depression, anxiety, and stress) during pregnancy, including underlying mechanisms and implications for integrated care delivery. The findings will inform the evidence-based integration of oral health screening into prenatal care and establish a foundation for developing comprehensive approaches that address both oral and psychological needs during pregnancy [28].

## 2. Materials and Methods

This rapid review follows the guidelines outlined in the Rapid Review Guidebook [29] and utilizes the PRISMA-RR checklist [30] to synthesize evidence. The decision to conduct a rapid review rather than a systematic review was based on several factors: (1) the need to provide timely evidence synthesis for clinical practice decisions, (2) the emergence of new evidence in this field requiring regular updates, and (3) the focused nature of our research question. While maintaining methodological rigor, this approach allowed completion within 12 weeks to inform time-sensitive practice and policy decisions.

### 2.1. Research Framework

The investigation and search strategy development were guided by the Population, Concept, Context (PCC) framework [29]. The study included pregnant women at any stage of gestation and women up to one year postpartum in various healthcare settings. The researchers examined oral health conditions (specifically periodontal disease and dental caries) alongside specific psychological conditions (anxiety, depression, and stress-related outcomes). The context addressed prenatal and postnatal care in healthcare settings globally from January 2000 to November 2024. The framework was defined as outlined in Table 1.

### 2.2. Search Strategy

The researchers conducted comprehensive searches between 27 August and 6 September 2024, in OVID Medline, CINAHL, and PsycINFO databases. The search strategy used the following key concepts and related terms:

1. Pregnancy/postpartum terms: “pregnant”, “pregnancy”, “antenatal”, “perinatal”, “prenatal”, “postpartum”, “postnatal”;
2. Oral health terms: “oral health”, “dental health”, “periodontal disease”, “gingivitis”, “dental caries”, “tooth decay”;
3. Psychological terms: “anxiety”, “depression”, “stress”, “psychological distress”, “mental health”;
4. Healthcare setting terms: “prenatal care”, “antenatal care”, “maternal health services”.

These terms were combined using appropriate Boolean operators and adapted for each database’s specific requirements. The researchers supplemented database searches with a Google Scholar search, examining the first 200 results using modified search strings adapted for the platform’s search capabilities. Appendix A presents the full search strategy for OVID Medline.

### 2.3. Eligibility Criteria

Studies qualified for inclusion by examining pregnant populations at any gestational stage or within one year postpartum and by investigating oral health conditions and specific psychological conditions (anxiety, depression, and stress). Observational studies, including cohort, case–control, and cross-sectional designs, along with interventional studies such as randomized controlled trials and quasi-experimental designs, were included. Furthermore, the team considered systematic reviews and meta-analyses that addressed the research questions. All studies reported quantifiable outcomes in peer-reviewed English-language journals from January 2000 onwards. Studies were excluded if they: (1) focused solely on non-pregnant populations, (2) examined oral health or psychological conditions in isolation, (3) used non-empirical methodologies including qualitative studies, or (4) did not report specific measurable outcomes for both oral health and psychological conditions.

### 2.4. Study Selection

Two independent reviewers (AA, SR) conducted the screening process using Covidence (2022 software) [31]. The process involved initial title screening, followed by abstract review and full-text assessment against eligibility criteria. The reviewers resolved all disagreements through structured discussion, achieving consensus without requiring third-party arbitration. Covidence software maintained a complete audit trail of screening decisions and exclusion reasons.

### 2.5. Quality Assessment and Data Analysis

While rapid reviews often modify quality assessment procedures to meet time constraints, we maintained methodological rigor by conducting comprehensive data extraction and synthesis; using standardized data extraction forms; employing dual review for key decisions and maintaining transparent documentation of all methodological choices. This approach allowed for thorough analysis while meeting the 12-week completion timeline necessary for informing current practice needs.

### 2.6. Data Extraction

The team developed and pilot-tested a standardized data extraction form to ensure comprehensive and consistent data collection. One reviewer extracted data from all included articles. The form captured bibliographic information, study characteristics such as design, setting, and sample size, participant demographics, interventions or exposures, outcome measures including primary and secondary measures, key findings related to oral–mental health relationships, and study limitations.

### 2.7. Data Synthesis

The narrative synthesis adhered to the guidelines set forth by Popay et al. (2006) [32], organizing the findings into four sequential steps. The researchers characterized the included studies by examining their research design, methodological approach, and population characteristics. Next, the team analyzed assessment methods for both oral and psychological health measures, examining the validity and reliability of the measurement tools. Third, the team synthesized findings across three key domains: the prevalence of concurrent oral–mental health conditions, the pathways linking oral and mental health outcomes, and the effectiveness of interventions. The researchers identified research gaps and methodological limitations in the current evidence base. The structured approach allowed for systematic evidence synthesis, even with significant differences in study designs and outcome measures.

### 2.8. Study Limitations

The rapid review methodology required a few adaptations from conventional systematic review approaches. The team prioritized comprehensive data extraction and synthesis, focusing on urgent practice needs while ensuring methodological rigor was maintained over formal quality assessment. The restriction to English-language studies may have led to the exclusion of relevant international research, while the compressed timeframe constrained the depth of analysis achievable for each study. However, this approach aided the feasibility of this rapid review. The limitations informed the interpretation of findings and shaped recommendations for future research.

### 2.9. Ethical Considerations

Being a rapid review of literature, the review did not require formal ethical approval, yet thorough research integrity standards were upheld throughout the process. Clear reporting and thorough data extraction received focused attention. The team integrated patient-centered outcomes to demonstrate their commitment to relevant and applicable findings. The researchers incorporated patient priorities from existing literature into their research questions and outcome measures, ensuring the review’s relevance to clinical practice.

## 3. Results

The rapid review revealed compelling evidence for associations between oral health conditions and psychological states (anxiety, depression, and stress) during pregnancy, manifesting through behavioral, physiological, and intergenerational pathways.

### 3.1. Study Characteristics

**Figure 1:**
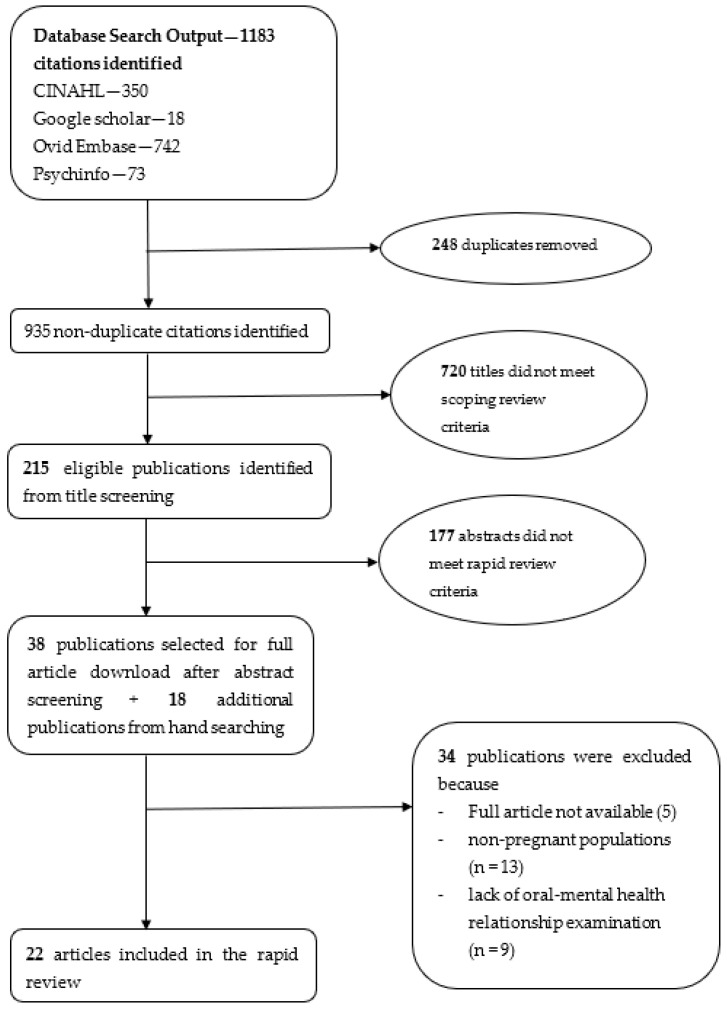
PRISMA Flow Chart for the Scoping Review.

From 1201 records (1183 database searches, 18 hand-searched), 22 studies met inclusion criteria with most studies (*n* = 14) published after 2020. Figure 1 presents the PRISMA flow diagram detailing our search and selection process.

The characteristics and findings of included studies are summarized in Table 2 and Table 3. Most studies employed cross-sectional designs (59.1%) and were predominantly conducted in the Middle East (31.8%) and North America (22.7%). Detailed study characteristics and findings are presented in Table 2.

The studies varied in their focus populations, with most targeting pregnant women exclusively, while some included comparative groups of non-pregnant women or extended to mother–child pairs. Sample sizes ranged from 46 to 790,758 participants: thirteen studies had fewer than 500 participants [7,9,10,13,33,34,35,36,37,38,39,40,41,42], eight studies included 500–2500 participants [41,43,44,45,46,47,48,49], and one large cohort study analyzed 790,758 mother–child pairs [50]. The most recent studies (2021–2024) showed a trend toward larger sample sizes, with five of the eight studies from this period including more than 500 participants [43,44,45,46,48,50].

**Table 3:** Characteristics and Key Findings of Studies Examining Oral Health and Mental Well-being During Pregnancy.

Author(s) and Year	Location	Study Design	Sample Size	Population Characteristics	Oral Health Measures/Scales	Mental Health Measures/Scales	Key Findings	Limitations
An et al. (2024) [48]	West Virginia and Pittsburgh, USA	Longitudinal	1172	Pregnant women	Self-reported toothbrushing and flossing frequency questionnaire	GEE approach for depression and stress assessment	Depression and stress negatively related to oral self-care	Self-reported measures
Gastmann et al. (2024) [36]	Brazil	Prospective	60	Pregnant and non-pregnant women needing root canal	Numerical pain scale (NPS); clinical assessment of dental pain	Dental Anxiety Scale (DAS); Pain Catastrophizing Scale (PCS)	No difference in pain, anxiety between pregnant/non-pregnant women	Limited pain assessment
Gokturk et al. (2024) [37]	Turkey	Comparative	87	Pregnant and non-pregnant women with/without gingivitis	Plaque index (PI); gingival index (GI); probing pocket depths (PPDs); GCF samples	Salivary cortisol levels; stress hormone analysis (ELISA)	Periodontal therapy improved stress markers in non-pregnant women only	Small sample size
Nazir et al. (2022) [46]	Saudi Arabia	Cross-sectional	780	Pregnant women	Dental attendance records	GAD-7; Modified Dental Anxiety Scale (MDAS)	Highest anxiety in third trimester	Self-reported data
Kim et al. (2022) [45]	Korea	Cross-sectional	1096	Pregnant women aged 19–55 years	Subjective oral health status questionnaire	Patient Health Questionnaire-9 (PHQ-9)	Higher subjective oral health status associated with decreased depression	Limited psychological factors
Cademartori et al. (2022) [44]	Brazil	Cross-sectional	2496	Pregnant women from birth cohort	DMF-T index; self-perceived oral health questionnaire	Edinburgh Postnatal Depression Scale (EPDS)	Dental caries’ effect on depression mediated by oral health self-perception	Limited to specific cohort
AlRatroot et al. (2022) [43]	Saudi Arabia	Cross-sectional	825	Pregnant women from hospitals	WHO Oral Health Survey; dental attendance patterns	Modified Dental Anxiety Scale (MDAS)	90.9% prevalence of dental anxiety	Limited geographic region
Alhareky et al. (2021) [33]	Saudi Arabia	Cross-sectional	199	Mother–child pairs	dmft/DMFT indices (WHO criteria)	Modified Dental Anxiety Scale (MDAS)	Higher maternal anxiety associated with more child caries	Small sample size
Yarkac et al. (2021) [39]	Turkey	Comparative	60	Pregnant and non-pregnant women with gingivitis	Periodontal indices (PI, GI, PPD); GCF samples	Perceived Stress Scale (PSS)	Worse periodontal outcomes in pregnant women	Small sample size
Auger et al. (2020) [50]	Quebec, Canada	Longitudinal cohort	790,758	Mother–child pairs in Quebec	Childhood dental caries (ICD codes)	Maternal mental disorders (diagnostic codes)	More caries in children of mothers with mental disorders	Administrative data limits
Ahmed et al. (2017) [40]	Saudi Arabia	Cross-sectional	438	Pregnant women	Oral health problems self-report checklist	Perceived Stress Scale (PSS-10)	33.4% high perceived stress	Self-reported measures
Luo et al. (2017) [41]	China	Cross-sectional	502	Pregnant women from outpatient clinic	Oral Health Impact Profile (OHIP-14)	Dental anxiety questionnaire	26.7% had dental anxiety; negative correlation between dental anxiety and oral-health-related quality of life	Self-reported measures
McNeil et al. (2016) [49]	Northern Appalachia, USA	Cross-sectional	681	Caucasian pregnant women	Gingivitis scale; ORI; DMFT index	Center for Epidemiologic Studies Depression Scale (CES-D)	Depressed women had poorer oral health	Limited to Caucasian women
Park et al. (2016) [38]	South Korea	Cross-sectional	129	Pregnant women	Community Periodontal Index (CPI)	Depression scale; stress scale	Periodontal disease associated with stress, depression	Small sample size
Seraphim et al. (2016) [7]	Brazil	Cross-sectional	96	Pregnant women at 5–7 months’ gestation	Community Periodontal Index (CPI)	Perceived Stress Scale (PSS); salivary cortisol	Higher perceived stress in periodontitis/gingivitis groups	Limited public health system data
Silveira et al. (2016) [13]	USA	Cross-sectional	402	Pregnant women	Self-reported tooth loss; dental visit frequency	PHQ-8; self-reported anxiety/depression diagnosis	3.30 times greater odds of tooth loss with anxiety	Self-reported data
[9] Gümüş et al. (2015) [9]	Turkey	Case–control	187	Pregnant, postpartum, controls	Periodontal disease severity indices	Oxidative stress markers	Higher oxidative stress in pregnant women	Clinical value not evaluated
Tolvanen et al. (2013) [42]	Finland	Longitudinal	254	Pregnant mothers and fathers	Modified Dental Anxiety Scale	EPDS; Anxiety Subscale SCL-90	Dental fear increased over pregnancy	Not specified in data
Yarkac et al. (2018) [10]	Turkey	Comparative	60	Pregnant and non-pregnant women with gingivitis	Plaque index; gingival index; GCF samples	Salivary chromogranin A (CgA); ELISA	Periodontal therapy improved status in both groups	Limited stress markers
Arteaga-Guerra et al. (2010) [34]	Colombia	Observational	46	Pregnant women	Periodontal examination protocol	Stress scale	Combined periodontitis and stress increased LBW risk	Small sample size
Esa et al. (2010) [35]	Malaysia	Cross-sectional	407	Antenatal mothers	D(3cv) MFS (dental decay) index	Dental Fear Survey (DFS)	Positive association between anxiety and decay	Single examiner bias
Horton et al. (2010) [47]	USA	Prospective cohort	1020	Pregnant women <26 weeks	Periodontal disease classification	Not specified	No evidence of oxidative stress mediation	Limited 236–266 longitudinal data

DMFT: Decayed, Missing, Filled Teeth; EPDS: Edinburgh Postnatal Depression Scale; GAD-7: Generalized Anxiety Disorder-7; GCF: Gingival Crevicular Fluid; MDAS: Modified Dental Anxiety Scale; PHQ: Patient Health Questionnaire; PSS: Perceived Stress Scale; WHO: World Health Organization; CPI: Community Periodontal Index; ORI: Oral Hygiene Index.

### 3.2. Assessment Methods

Psychological status assessment utilized validated instruments including the Modified Dental Anxiety Scale (MDAS) [33,43,46], Edinburgh Postnatal Depression Scale (EPDS) [42], and the Patient Health Questionnaire (PHQ-8/9) [13,45]. Studies measuring stress utilized the Perceived Stress Scale [7,40] alongside biological markers such as salivary cortisol [7,9]. Oral health evaluation combined clinical examinations using DMFT indices and WHO criteria with self-reported measures [51]. Most studies (n = 15) used validated mental health scales, while oral health assessment methods showed greater heterogeneity, ranging from comprehensive clinical examinations to basic self-report questionnaires.

### 3.3. Behavioral Pathways and Access Barriers

Dental anxiety emerged as a significant barrier to oral healthcare use, affecting 90.9% of pregnant women in one study [43] and significantly reducing dental attendance [13]. Dental anxiety’s effects emerged through three mechanisms: (1) direct effects on behavior: women who experienced anxiety showed almost three times greater odds of avoiding dental care [13], (2) psychological amplification: depression was associated with lower oral health self-efficacy (β = −0.28, *p* < 0.001) and heightened dental anxiety (β = 0.47, *p* < 0.001) [48], and (3) effects mediated by stress: stressful life events led to an increase in both poor oral health (OR: 1.56) and unmet dental needs (OR: 1.86) in one study [52].

### 3.4. Physiological Mechanisms

Several studies demonstrated associations between oral health and stress responses [7,10,37]. The findings included correlations between elevated blood glucose and insulin resistance in pregnant women with periodontitis [7] and higher oxidative stress markers in pregnancy-associated gingivitis [37]. Evidence also indicates associations in treatment outcomes as periodontal therapy was associated with reduced inflammation and stress markers [10]. Pregnancy-specific modifications to inflammatory responses, evidenced by differential treatment outcomes in pregnant versus non-pregnant women, were also reported [10].

### 3.5. Intergenerational Transmission

Data suggested associations between maternal oral–psychological status and child health outcomes [33,50]. A retrospective cohort study (n = 790,758) examining children from birth to age 12 revealed an association between maternal psychological conditions and increased caries risk in children, with a cumulative incidence of 62.9 per 1000 children for maternal psychological conditions versus 31.4 per 1000 for no maternal disorder by age 12 [50]. Another study reported correlations between maternal dental anxiety and children’s untreated decay [33].

### 3.6. Research Gaps and Limitations

The reviewed studies present several methodological limitations that affect the interpretation and generalizability of findings. The predominance of cross-sectional designs [13,41,49] limits our understanding of causal relationships between mental health and oral health outcomes during pregnancy. Only three studies [42,48,50] used longitudinal approaches and none used qualitative approaches. The wide variation in sample sizes creates challenges in comparing findings and establishing reliable effect estimates. Geographic concentration on specific regions, particularly Saudi Arabia [43,46] and the United States [13,49], also limits global applicability of the findings. Methodological heterogeneity further complicates the synthesis of evidence. While some researchers used validated instruments such as MDAS [43,46], others relied on non-standardized or self-reported measures [40,41]. Studies investigating biological markers also typically involved small samples [7,10] and rarely included clinical oral health examinations, relying instead on self-reported oral health status [41].

## 4. Discussion

The rapid review reveals compelling evidence for bidirectional associations between oral and psychological states (anxiety, depression, and stress) during pregnancy, highlighting significant implications for maternal and child health outcomes [1,2]. Three primary mechanisms emerge from the evidence. First, biological mechanisms play a crucial role where stress hormones (particularly cortisol) modulate immune responses and gingival inflammation [7,10]. Psychological stress affects oral immune function and wound healing [9], while periodontal inflammation shows reciprocal relationships with systemic stress responses [7,10,39]. Importantly, pregnancy hormones may amplify these biological interactions [2,3].

Second, behavioral mechanisms significantly influence outcomes. Dental anxiety acts as a barrier to seeking dental care [13,35,36,41,43,46], while depression reduces oral health self-efficacy and self-care behaviors [11,13]. Stress impacts adherence to oral hygiene routines [53], creating a cycle of deteriorating oral health [19,20,21]. Third, psychosocial mechanisms underlie many observed relationships. Social determinants affect access to both dental and psychological care, while cultural factors influence care-seeking behaviors [54]. Economic barriers often limit access to preventive services, exacerbating health disparities [19,20,21]. These findings extend current understanding beyond previously established general population links in the general population [17,55] and highlight pregnancy as a critical period for integrated healthcare interventions.

The biological relationships between periodontal conditions and stress biomarkers [7,9,10,39] provide biological plausibility for oral–mental health interactions during pregnancy. This is further supported by recent microbiome research showing distinct oral bacterial profiles in pregnant women with different levels of anxiety and depression symptoms [18]. Studies suggest that periodontal therapy on oral health parameters and stress markers indicate potential therapeutic benefits that extend beyond the improvement of oral health [10]. The modified inflammatory responses observed in pregnant women indicate a need for treatment protocols that are specific to pregnancy [56]. The study findings align with evidence suggesting associations between periodontal health and pregnancy outcomes [2] and suggest that oral health interventions could provide multiple benefits during pregnancy.

The evidence for intergenerational transmission of oral health risks is particularly compelling [33,50]. Research suggests associations between maternal psychological conditions and increased risk of caries, indicating that pregnancy serves as a crucial intervention window for breaking cycles of poor health outcomes. This risk is further amplified by direct bacterial transmission mechanisms, where cariogenic bacteria are more readily transferred from mothers with untreated decay to their children [57]. Studies have demonstrated specific maternal–child bacterial colonization patterns [58], emphasizing the importance of both preventive and operative dental care during pregnancy to reduce children’s future caries risk. This finding extends previous research on the relationships between maternal and child oral health [3] and provides additional justification for the integration of oral health into prenatal care [11,12].

### Implications for Practice

For effective implementation of integrated care, we recommend several key approaches. Enhanced communication and health literacy form the foundation of improved care delivery [59,60]. This includes developing culturally competent communication strategies, providing oral health education in accessible formats, and building trusting, non-judgmental provider–patient relationships. Provider training in effective communication techniques is essential for successful implementation [61]. Furthermore, community-based approaches represent another crucial element of integrated care. Integration of community health workers into care teams leverages their cultural and linguistic competencies [62]. These workers can utilize existing community networks for health education and help establish community-based support systems that enhance care delivery and outcomes.

Healthcare providers should implement combined oral–psychological health screening during routine prenatal visits [14,15]. This approach facilitates early identification of interrelated conditions and enables timely intervention. Healthcare professionals’ comprehensive interprofessional education begins in their foundational training programs. This should include joint learning opportunities where dental, medical, nursing, and mental health students train together to understand the interconnections between their disciplines and develop collaborative care approaches.

Implementation of integrated care requires both structural and cultural changes. Healthcare systems should invest in shared electronic health records and care coordination platforms that facilitate communication between providers. Regular case conferences and team meetings can help build relationships between different specialists. Financial incentives and reimbursement models should be aligned to support integrated care delivery. Additionally, healthcare organizations should foster a culture of collaboration by recognizing and rewarding cross-disciplinary initiatives.

The review presents strong evidence for the associations between oral and psychological health during pregnancy, yet it acknowledges several research gaps that need attention. Future studies should adopt longitudinal designs to better establish causality, include diverse geographic populations, and utilize standardized assessment protocols. The investigation of intervention effectiveness and cost-effectiveness of integrated care models can strengthen the evidence base for implementation [28].

## 5. Conclusions

This rapid scoping review establishes strong interconnections between oral and psychological states (anxiety, depression, and stress) during pregnancy, highlighting implications for maternal and child health outcomes. Evidence supports the implementation of pregnancy-specific integrated screening protocols, the development of targeted interventions, and the establishment of coordinated care pathways within existing prenatal care communities. Systematic changes in clinical practice and health policy can help break intergenerational cycles of poor health outcomes. Future research will focus on the development and evaluation of standardized assessment protocols and integrated care models across diverse populations. The implementation of these evidence-based recommendations could significantly enhance maternal and child health outcomes by providing comprehensive prenatal care that addresses both oral and psychological needs.

## Figures and Tables

**Table 1:** Research Framework.

Component	Description
Population	- Pregnant women at any stage of pregnancy - Women in the postpartum period (up to one year after childbirth)
Concept	- Oral health status and conditions (e.g., periodontal disease, dental caries) - Mental health status and conditions (e.g., depression, anxiety) - Stress levels and stress-related outcomes - Relationship between oral health and mental health - Impact of stress on both oral and mental health
Context	- Prenatal care settings - Postnatal care settings - Healthcare settings relevant to pregnancy and postpartum - Timeframe: From pregnancy through the first year postpartum - Geographical context: Studies from any country or cultural setting

**Table 2:** Distribution of Included Studies by Design and Geographic Region.

Study Characteristics	Number of Studies (n)	Percentage (%)
Study Design		
Cross-sectional	13	59.1
Comparative	3	13.6
Longitudinal cohort	3	13.6
Prospective	2	9.1
Case–control	1	4.5
Geographic Region		
Middle East	7	31.8
North America	5	22.7
South America	3	13.6
Asia Pacific	4	18.2
Europe	2	9.1
Other	1	4.5
Total	22	100

## Data Availability

No new data were created or analyzed in this study. Data sharing is not applicable to this article.

## Appendix A

OVID EMBASE Search Strategy

1. exp pregnancy/
2. (pregnant or pregnancy or expectant or maternal or antenatal or perinatal or prenatal). kw.
3. exp puerperium/
4. (postpartum or postnatal or puerperal or “new mother*”).ti,ab,kw.
5. exp pregnancy care/or exp antenatal care/or exp postnatal care/
6. (prenatal care or antenatal care or postnatal care or postpartum care or “after-birth care”). kw.
7. or/1–6
8. exp tooth disease/or exp mouth disease/
9. (oral health or dental health or periodontal disease or gingivitis or dental caries or tooth decay or oral hygiene).ti,ab,kw.
10. or/8–9
11. exp mental health/or exp depression/or exp anxiety/or exp stress/
12. (mental health or psychological health or depression or anxiety or stress or mood disorder* or perinatal depression or postpartum depression).ti,ab,kw.
13. or/11–12
14. exp health care/
15. (healthcare or health service* or maternal health service* or dental service* or mental health service*).ti,ab,kw.
16. or/14–15
17. 7 and 10 and 13 and 16
18. limit 17 to (english language and yr = “2000–Current”)
